# A retrospective study of tracheal collapse in small-breed dogs: 110 cases (2022–2024)

**DOI:** 10.3389/fvets.2024.1448249

**Published:** 2024-08-14

**Authors:** Mi-Rae Kim, Se-Hoon Kim, Min-Ok Ryu, Hwa-Young Youn, Ji-Hye Choi, Kyoung-Won Seo

**Affiliations:** ^1^Laboratory of Veterinary Internal Medicine, Department of Veterinary Clinical Science, College of Veterinary Medicine, Seoul National University, Seoul, Republic of Korea; ^2^Department of Veterinary Medical Imaging, College of Veterinary Medicine, Seoul National University, Seoul, Republic of Korea

**Keywords:** tracheal collapse, fluoroscopy, chronic cough, tracheobronchomalacia, dogs

## Abstract

**Background:**

The grade of tracheal collapse (TC) is assessed by the diameter of the narrowed lumen. However, studies on the relationship between TC grade and clinical symptom severity are lacking.

**Objectives:**

To investigate the clinical characteristics of small-breed dogs diagnosed with TC and determine if fluoroscopic grading correlates with cough severity.

**Methods:**

We retrospectively reviewed medical records from 2022 to 2024. TC diagnosis was confirmed using fluoroscopic examination. Multiple linear regression was employed to investigate factors influencing cough severity, with a significance level set at *p* < 0.05.

**Results:**

A total of 132 dogs with TC were identified, of which 22 were excluded. The final cohort consisted of 110 dogs, aged between 2–19 years, with no significant sex differences. The majority (97.2%) of dogs had a BCS of ≥4. Among the top four breeds (Maltese, Pomeranian, Poodle, and Chihuahua), the most severe collapse was observed in the carinal region. The grade of collapse on fluoroscopy was mostly related to high BCS (*p* < 0.007) and low body weight (*p* < 0.001). However, interestingly, fluoroscopic findings of collapse location and grade did not correlate with cough severity (*p* = 0.350). Notably, clinical symptoms improved in 86.6% of cases following interventions such as weight reduction, environmental changes, and pharmacotherapy.

**Conclusions and clinical relevance:**

In small-breed dogs, the severity of cough was not associated with the region or grade of TC diagnosed by fluoroscopy.

## 1 Introduction

Tracheal collapse (TC) is a common cause of chronic cough in dogs (1–5), characterized by dorsoventral flattening of the tracheal rings and laxity of the dorsal tracheal membrane (1, 6). Previous studies indicate that TC predominantly affects miniature, toy, and small-breed dogs—such as Yorkshire Terriers, Pomeranians, Pugs, Poodles, and Chihuahua (7, 8) dogs—with rare occurrences in large-breed dogs and cats (9, 10). Most affected dogs were middle-aged or older, with no significant sex predilection. Primary or congenital abnormalities in tracheal cartilage and secondary factors exacerbating clinical signs may be key etiological factors, particularly in small breeds (11).

The pathophysiology of cough in dogs with TC is multifactorial and poorly understood, and some dogs remain asymptomatic until they reach older age (12). Secondary triggers of clinical symptoms include airway irritants, chronic bronchitis, respiratory infections, endotracheal intubation, laryngeal paralysis, obesity, and changes in the elastic fiber of the dorsal tracheal membrane and annular ligament (8, 11, 13). Once coughing begins, dynamic airway collapse can lead to chronic tracheal mucosa inflammation, further aggravating the cough (14). The severity of TC is determined by the extent of luminal diameter reduction in the affected region (2). In humans, the gold standard for diagnosing airway collapse or tracheobronchomalacia (TBM) is flexible bronchoscopy (15–17). Similarly, bronchoscopy is a valuable diagnostic tool for airway collapse in dogs (1, 3). However, because bronchoscopy requires anesthesia, it limits the assessment of airway changes during coughing (5). Conversely, fluoroscopy is a dynamic imaging technique that can evaluate multiple regions of collapse during all phases of respiration, including coughing (8). Studies have shown that fluoroscopic findings during coughing correlate 87–100% with airway collapse identified by bronchoscopy (4, 5). Thus, further studies are needed to investigate the correlation between the degree of collapse on fluoroscopy and clinical symptoms in dogs with TC.

It is well established that the incidence of TC is higher in small breeds (7, 8). However, no studies have specifically compared the characteristics of TC among different small-breed dogs. Thus, this study aimed to investigate the clinical characteristics of small-breed dogs with TC and determine whether fluoroscopic classification correlates with the severity of coughing.

## 2 Materials and methods

### 2.1 Study design

The retrospective review of medical records was authorized by our institution, and the client consented to the use of the records.

### 2.2 Animals

All dogs in this study were referred between January 2022 and January 2024 to our hospital in the Republic of Korea. Inclusion criteria were dogs diagnosed with TC based on fluoroscopic findings and weighing <10 kg; however, those whose chief complaint was not coughing on the day of diagnosis, despite fluoroscopic evidence of TC, were excluded. Additionally, dogs with comorbid conditions known to cause coughing—such as lung parenchymal diseases, oronasal diseases, and cardiac diseases—were excluded if their conditions were not well-controlled.

### 2.3 Data collection and clinical evaluation

The medical records of the dogs included in this study were obtained on the day of their visits. The following information was collected: signalment, clinical signs, number of coughs after tracheal stimulation, radiographic and fluoroscopic images, comorbidities, and treatment options. Tracheal stimulation was performed by applying gentle pressure to the cervical region of the dog, and the number of coughs was recorded within a 5-min period. Cough severity upon tracheal stimulation was categorized from medical records as mild (0–1 times), moderate (2–4 times), or severe (≥5 times). Comorbidities assessed included myxomatous mitral valve disease [MMVD, according to the American College of Veterinary Internal Medicine (ACVIM) staging] and pulmonary hypertension (PH). All patients underwent radiological and fluoroscopic examinations, with some also receiving echocardiography, which was performed and interpreted by radiologists. The MMVD stage was determined by an internist based on clinical presentation and echocardiographic findings in accordance with the ACVIM consensus (18). PH was diagnosed by an internist based on clinical presentation and echocardiographic evidence (19). Notably, only patients with well-controlled MMVD and PH were included in this study.

### 2.4 Diagnosis of TC

**Figure 1:**
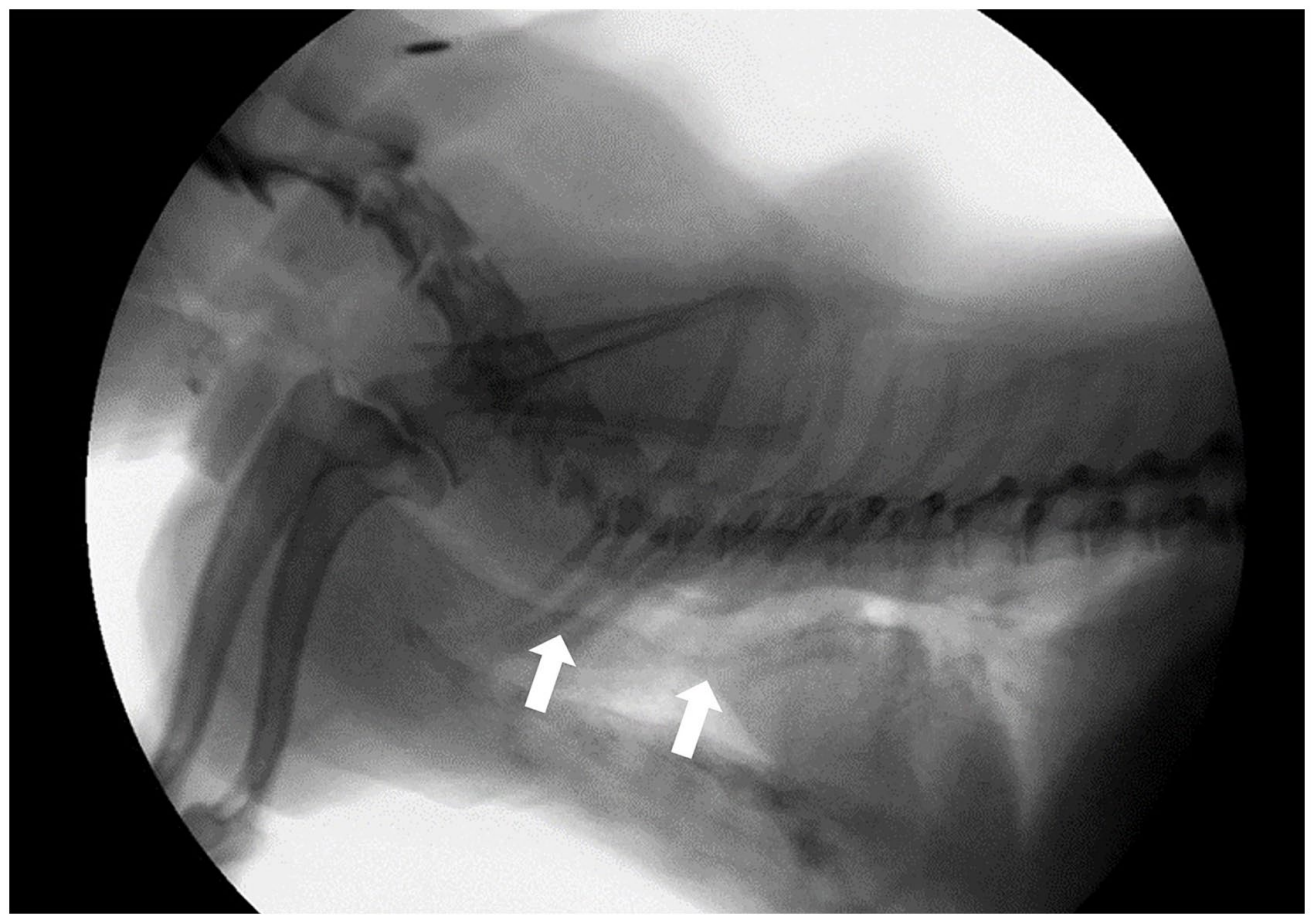
Fluoroscopic image of a dog in the right lateral position during a breathing phase. White arrows indicate; tracheal kinking.

All dogs in the study were evaluated for clinical signs and underwent fluoroscopy to diagnose TC (8). Radiographic studies were also performed. Clinical signs assessed included chronic goose-honking cough, exercise intolerance, cyanosis, respiratory distress, and syncope. A commercially available digital radiography system (EVA-HF 525; ECORAY, Seoul, Republic of Korea) was used to obtain right lateral and dorsoventral thoracic radiographs during inspiration. Based on the assumption that the fluoroscopic diagnosis was accurate, we analyzed whether TC could be diagnosed from the inspiratory radiographs. Fluoroscopy (SPINEL 3G; GEMSS Healthcare Corporation, Paju, Republic of Korea) was conducted during both the normal breathing and cough (forced expiration) phases, with the dogs positioned in right lateral recumbency. Collapse was defined as a reduction in lumen diameter of ≥25% on fluoroscopy. TC grades were assessed according to the degree of maximal collapse of the tracheal luminal diameter (0, 25, 50, 75, and 100%) based on the criteria developed by Tangner and Hobson (2, 20). Cervical lung lobe herniation (CLLH) grade was evaluated by positioning the patient in a humanoid view and comparing the herniated apical level of the lung lobe with the body of the cervical vertebra during the coughing phase (21). The main stem bronchi were assessed for the presence or absence of collapse during the cough phase, but no severity grade was assigned to them. Tracheal kinking was assessed based on the loss of the linear shape or presence of an undulating form of the trachea (Figure 1).

### 2.5 Assessment and scoring of coughing

Because no objective criteria have been established in veterinary medicine for assessing the severity of cough in dogs, the severity of cough was assessed using a cough score sheet from a previous study (22). The assessment was based on medical records and included the timing of the cough and circumstances that triggered it. Changes in cough severity were evaluated after 2–4 weeks of treatment. All cough scores were re-evaluated in random order by an internist who was blinded to the fluoroscopic results, and the two assessments were in agreement. The evaluation criteria are listed in Table 1.

**Table 1:** Cough scoring criteria.

	Score	Clinical correlation
Presence or absence	1	None
	2	Yes
Time of day	1	Dog coughs during the night/early morning
	2	Dog coughs continuously during the day
Situation	1	Dog coughs only during excitement
	2	Dog coughs at rest during the daytime
Change in cough after treatment	1	Cough disappeared
	2	Cough decreased but remained
	3	Unchanged or increased

### 2.6 Treatment

As this study was retrospective, the prescribed treatment was at the discretion of the attending veterinarian. Treatments prescribed at diagnosis were recorded, and cases were divided into the following subgroups: environmental changes with weight loss alone, bronchodilators, anti-inflammatory drugs, and antitussives, each used alone or in combination with two or more other treatments. Any additional treatments prescribed were also recorded.

### 2.7 Statistical analysis

Statistical analyses were performed using SPSS software (SPSS for Windows version 25.0; IBM, Chicago, IL). Descriptive statistics were reported as mean ± standard deviation (SD). Graphs were created using GraphPad Prism software (GraphPad Prism version 10.0.0; GraphPad Software, Inc., Boston, MA). Multiple linear regression was used to investigate factors influencing the severity of TC by location and factors influencing pretreatment cough scores in patients with TC weighing <10 kg. The effects of treatment on cough duration and circumstances were examined using McNemar’s test. Fisher’s exact test was used to assess changes in cough severity according to the number of medications taken and weight loss. Statistical significance was set at a *p*-value <0.05.

## 3 Results

Data were collected over a period of 2 years (2022–2024) by a single investigator. A total of 132 dogs were initially identified, with 22 subsequently excluded after a thorough case review. Reasons for exclusion included pulmonary infiltration (*n* = 7), pulmonary mass (*n* = 7), body weight (BW) >10 kg (*n* = 3), oronasal fistula (*n* = 2), heartworm (*n* = 1), heart base tumor (*n* = 1), and atrial septal defect (*n* = 1). Eventually, the final number of dogs included and analyzed in this study was 110/132 (83%).

Epidemiological data of the studied dogs—including age, sex, BW, breed, body condition score (BCS), comorbidities, clinical signs, and radiological identification of TC—are summarized in Table 2. The study population comprised 110 dogs, including 65 males (63 neutered and 2 intact) and 45 females (43 spayed and 2 intact). The mean ± SD age, BW, and BCS were 11.37 ± 3.26, 4.68 ± 1.92 kg, and 5.44 ± 1.32, respectively.

**Table 2:** Characteristics of the study population.

Characteristic		*n* (%)
Age (year)	Mean ± SD	11.37 ± 3.26
Sex	Male	2 (1.8%)
	Female	2 (1.8%)
	Neutered male	63 (57.3%)
	Spayed female	43 (39.1%)
BW (kg)	Mean ± SD	4.68 ± 1.92
BCS	Mean ± SD	5.44 ± 1.32
Breed	Maltese	34 (30.9%)
	Pomeranian	25 (22.7%)
	Poodles	17 (15.5%)
	Chihuahua	8 (7.3%)
	Yorkshire	6 (5.5%)
	Bichon Frise	5 (4.5%)
	Mix Breed	5 (4.5%)
	Shih Tzu	3 (2.7%)
	Schnauzer	2 (1.8%)
	Spitz	2 (1.8%)
	Cocker Spaniel	1 (0.9%)
	Miniature Pinscher	1 (0.9%)
	Pompitz	1 (0.9%)
Comorbidity (MMVD)	B1	25 (22.7%)
	B2	28 (25.5%)
	Cc	8 (7.3%)
	None	49 (44.5%)
Comorbidity (PH)	Mild	18 (16.4%)
	Moderate	9 (8.2%)
	Severe	2 (1.8%)
	None	81 (73.6%)
Clinical sign^a^	Chronic cough	108 (98.1%)
	Cyanosis	11 (10.0%)
	Respiratory distress	10 (9.1%)
	Exercise intolerance	6 (5.5%)
	Syncope	9 (8.2%)
TC confirmed on radiographically	Yes	71 (64.5%)
	No	39 (35.5%)

Age, sex, BW, BCS, breed, comorbidities, clinical signs, and whether TC was confirmed radiographically in study population. BCS, body condition score; BW, body weight; MMVD, myxomatous mitral valve disease; PH, pulmonary hypertension; SD, standard deviation; TC, tracheal collapse.

a Multiple response.

Thirteen dog breeds were included in this study, with the most common being Maltese (34 dogs, 30.9%), followed by Pomeranian (25 dogs, 22.7%), Poodle (17 dogs, 15.4%), Chihuahua (8 dogs 7.2%), Yorkshire Terrier (6 dogs, 5.4%), Bichon Frise (5 dogs, 4.5%), mixed breed (5 dogs, 4.5%), Shih-Tzu (3 dogs, 2.7%), Schnauzer (2 dogs, 1.8%), Spitz (2 dogs, 1.8%), Miniature Pinscher (1 dog, 0.9%), Cocker Spaniel (1 dog, 0.9%), and Pompitz (1 dog, 0.9%).

Comorbidities observed included MMVD and PH; 55.4% of dogs had MMVD ACVIM stage B or higher, and 26% had PH [ACVIM stage B1 MMVD, 25 dogs (22.7%); stage B2, 28 dogs (25.5%); stage C, 8 dogs (7.3%); mild PH, 18 dogs (16.4%); moderate PH, 9 dogs (8.2%); and severe PH, 2 dogs (1.8%)]. Clinical symptoms at the time of diagnosis were assessed using a multiple-response format. Chronic cough was present in 98.1% of cases, cyanosis in 10.0%, dyspnea in 9.1%, exercise intolerance in 5.5%, and syncope in 8.2%. Assuming that the fluoroscopic diagnosis was precise, 64.5% of TC cases were confirmed by radiography.

**Figure 2:**
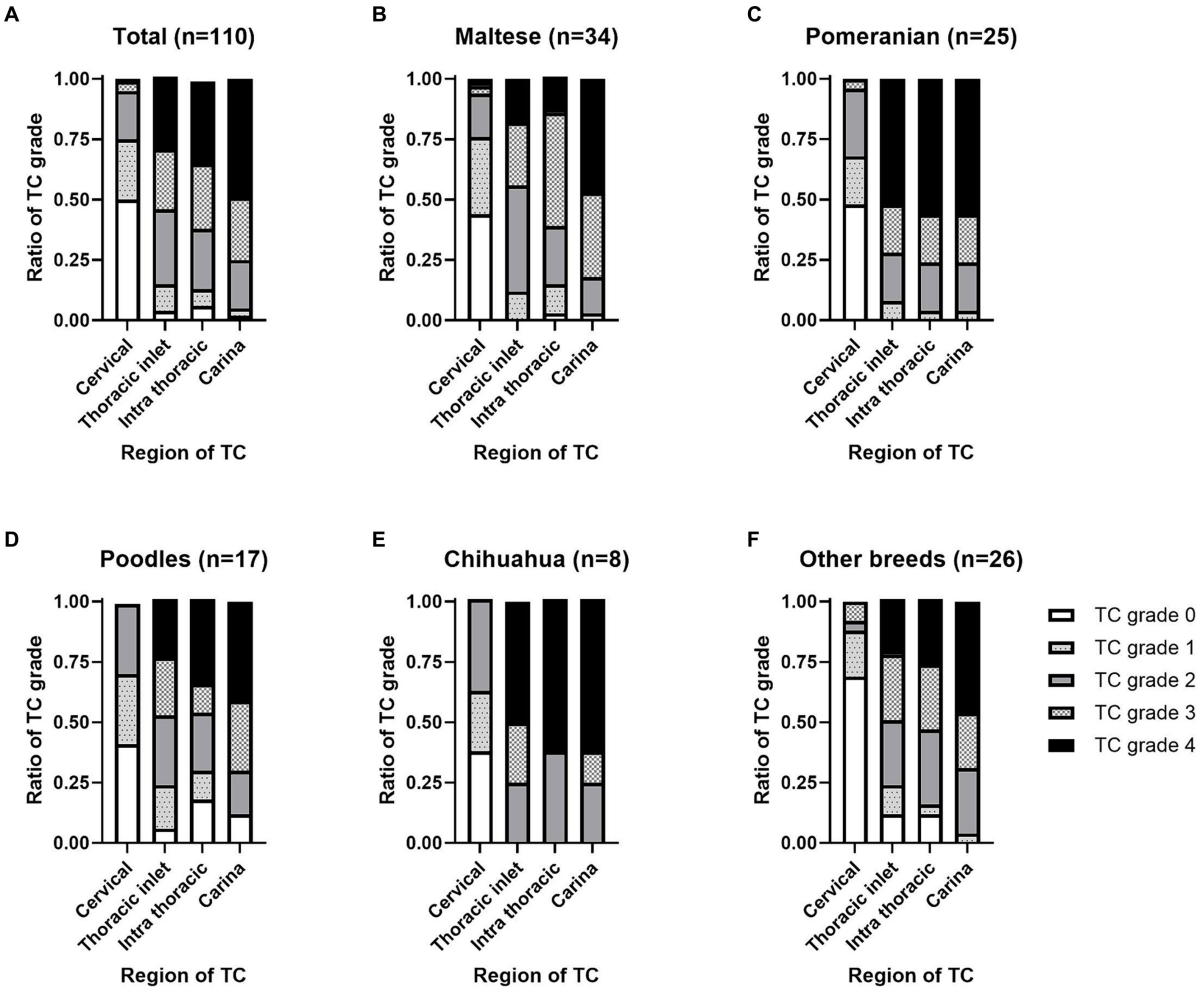
Location and grade of tracheal collapse (TC) occurrence on fluoroscopy by dog breed. An illustration of the distribution of TC among **(A)** the total dog population (*n* = 110). **(B)** Maltese (*n* = 34). **(C)** Pomeranians (*n* = 25). **(D)** Poodles (*n* = 17). **(E)** Chihuahuas (*n* = 8), and other breeds (*n* = 26). TC severity was assessed based on the percentage of maximal reduction in luminal diameter as follows: grade 0, 0%; grade 1, 25%; grade 2, 50%; grade 3, 75%; and grade 4, 100%.

**Figure 3:**
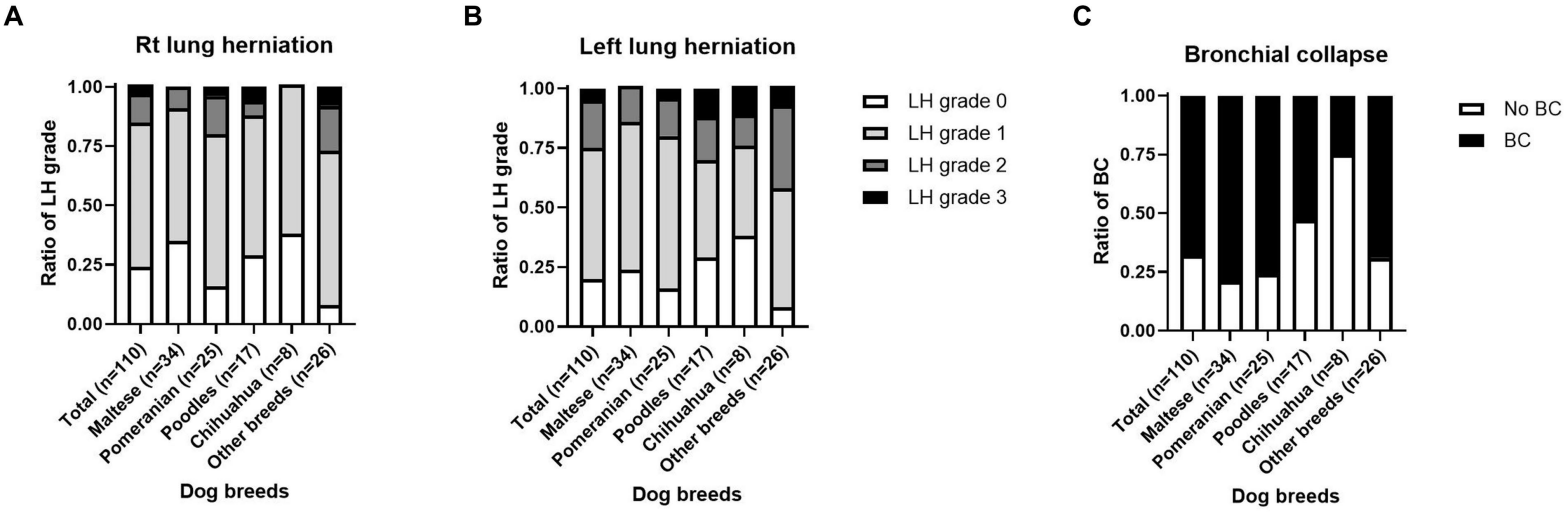
Grade of cervical lung lobe herniation (CLLH) and presence of bronchial collapse (BC) on fluoroscopy by dog breed. An image depicting the distribution of **(A)** right-sided CLLH, **(B)** left-sided CLLH, and **(C)** BC among different dog breeds. CLLH severity was evaluated by comparing the maximal herniation level of the cranial lung lobe to the cervical vertebral body in a humanoid view, with the following grades: grade 1, herniation at the 7th cervical vertebra; grade 2, herniation at the 6th cervical vertebra; and grade 3, herniation at the 5th cervical vertebra. The mainstem bronchus was assessed for the presence, partial presence, or absence of collapse. *p < 0.05, **p < 0.01.

Fluoroscopic images of all 110 dogs were obtained on the day of diagnosis. Fluoroscopic grading of the cervical, thoracic inlet, intrathoracic, and carinal regions was performed for all patients. The patients were categorized into the top four breeds and other breeds. Fluoroscopic TC grade, bilateral CLLH grade, and presence of bronchial collapse (BC) by breed are summarized in Figures 2, 3.

In total, TC was detected in the cervical (55 dogs, 50.0%), thoracic inlet (106 dogs, 96.3%), intrathoracic (103 dogs, 93.6%), and carinal (108 dogs, 98.1%) regions. The proportions of TC grades according to the location on the fluoroscopic images are shown in Figure 2A. Among the 34 Maltese cases, TC was detected in the cervical (19 dogs, 55.8%), thoracic inlet (34 dogs, 100%), intrathoracic (33 dogs, 97%), and carinal (34 dogs, 100%) regions. For the 25 Pomeranian cases, TC was detected in the cervical (13 dogs, 52.0%), thoracic inlet (25 dogs, 100%), intrathoracic (25 dogs, 100%), and carinal (25 dogs, 100%) regions. In the 17 Poodle cases, TC was detected in the cervical (10 dogs, 58.8%), thoracic inlet (16 dogs, 94.1%), intrathoracic (14 dogs, 82.3%), and carina (15 dogs, 88.2%) regions. Among the eight Chihuahua cases, TC was detected in the cervical (5 dogs, 62.5%), thoracic inlet (8 dogs, 100%), intrathoracic (8 dogs, 100%), and carina (8 dogs, 100%) regions. In the 26 cases of other breeds, TC was detected in the cervical (8 dogs, 50.0%), thoracic inlet (23 dogs, 96.3%), intrathoracic (23 dogs, 93.6%), and carinal (26 dogs, 100%) regions. The proportions of TC grades by location in the fluoroscopic images for each dog breed are illustrated in Figures 2B–F.

Of the 110 dogs, right CLLH was identified in 84 dogs (76.3%). Among these, right CLLH was observed in 22/34 Maltese (64.7%), 21/25 Pomeranians (84.0%), 12/17 Poodles (70.5%), and 5/8 Chihuahuas (62.5%). In other breeds, right CLLH was found in 24/26 dogs (92.3%). The proportions of right CLLH grades for each breed are shown in Figure 3A. Left CLLH was identified in 88 dogs (80.0%). Among these, left CLLH was observed in 26/34 Maltese (76.4%), 21/25 Pomeranians (84.0%), 12/17 Poodles (70.5%), and 5/8 Chihuahuas (62.5%). In other breeds, left CLLH was found in 24/26 dogs (92.3%). The proportions of left CLLH grades for each breed are shown in Figure 3B. BC was identified in 75 dogs (68.1%). Among these, BC was confirmed in 27/34 Maltese (79.4%), 19/25 Pomeranians (76%), 9/17 Poodles (52.9%), 2/8 Chihuahuas (25%), and 18/26 dogs (69.2%) of other breeds. The proportion of BC occurrence by breed is shown in Figure 3C.

Factors affecting the severity of TC according to location are presented in Table 3. Severity at the cervical site was influenced by BW (*B* = −0.226, *p* < 0.001), indicating that lower BW was associated with higher severity in the cervical region. The severity of the thoracic inlet area was influenced by BW (*B* = −0.213, *p* = 0.001) and BCS (*B* = 0.282, *p* = 0.002); this suggests that a lower BW and higher BCS were associated with increased severity at the thoracic inlet. The severity of the intrathoracic area was affected by BW (*B* = −0.249, *p* < 0.001), BCS (*B* = 0.334, *p* = 0.001), and left CLLH (*B* = 0.456, *p* = 0.046). Therefore, lower BW, higher BCS, and greater severity of the left CLLH were associated with increased severity in the intrathoracic region. For the carinal region, factors affecting severity included neutered status (*B* = 1.389, *p* = 0.006), BW (*B* = −0.187, *p* = 0.001), and BCS (*B* = 0.219, *p* = 0.006). Thus, being neutered and having a lower BW and higher BCS are all associated with increased severity in the carinal region. By contrast, age, comorbidities, right CLLH, BC, and cough count on tracheal stimulation were not associated with the severity of collapse at any site.

**Table 3:** Factors affecting collapse severity in the TC region.

	Cervical			Thoracic inlet			Intra thoracic			Carina		
	*B*	*β*	*p*	*B*	*β*	*p*	*B*	*β*	*p*	*B*	*β*	*p*
	1.089		0.163	1.759		0.051	1.426		0.119	1.113		0.156
Sex^a^	0.329	0.170	0.099	0.404	0.180	0.079	0.457	0.194	0.051	0.199	0.104	0.318
Neutered^a^	0.232	0.046	0.635	−0.469	−0.081	0.404	0.418	0.069	0.465	1.389	0.281	0.006^**^
Age	0.032	0.110	0.279	0.042	0.124	0.219	−0.007	−0.020	0.834	0.004	0.013	0.900
BW	−0.226	−0.447	<0.001^**^	−0.213	−0.365	0.001^**^	−0.249	−0.406	<0.001^**^	−0.187	−0.372	0.001^**^
BCS	0.031	0.040	0.690	0.282	0.314	0.002^**^	0.334	0.355	0.000^**^	0.219	0.284	0.006^**^
Comorbidity (MMVD)^a^	0.004	0.002	0.986	−0.318	−0.143	0.201	−0.216	−0.093	0.393	0.069	0.036	0.749
Comorbidity (PH)^a^	−0.369	−0.171	0.127	−0.231	−0.093	0.403	0.072	0.027	0.798	0.177	0.083	0.464
Rt CLLH	−0.106	−0.079	0.609	0.257	0.165	0.283	0.008	0.005	0.972	0.068	0.051	0.745
Lt CLLH	0.121	0.098	0.530	−0.039	−0.027	0.862	0.456	0.303	0.046^*^	0.094	0.076	0.629
Bronchial collapse^a^	−0.304	−0.148	0.134	0.291	0.122	0.210	−0.456	−0.183	0.056	−0.075	−0.037	0.710
TC confirmed on radiographically^a^	0.287	0.143	0.147	−0.094	−0.040	0.679	−0.005	−0.002	0.983	0.013	0.007	0.946
Cough count on tracheal stimulation 1^a^	−0.305	−0.132	0.215	−0.146	−0.055	0.606	0.092	0.033	0.748	−0.038	−0.017	0.876
Cough count on tracheal stimulation 2^a^	−0.078	−0.038	0.716	0.193	0.081	0.434	−0.067	−0.027	0.790	0.229	0.112	0.290
*F* (*p*)	2.357 (0.009^**^)			2.482 (0.006^**^)			2.996 (0.001^**^)			2.104 (0.021^*^)		
Adjusted *R*^2^	0.145			0.156			0.200			0.121		

BCS, body condition score; BW, body weight; CLLH, cervical lung lobe herniation; MMVD, myxomatous mitral valve disease; PH, pulmonary hypertension; TC, tracheal collapse. ^*^p < 0.05 and ^**^p < 0.01.

a Dummy variable: sex (1 = male, 0 = female), neutered (1 = yes, 0 = no), comorbidity (MMVD) (1 = yes, 0 = no), comorbidity (PH) (1 = yes, 0 = no), bronchial collapse (1 = yes, 0 = no), TC confirmed on radiographically (1 = yes, 0 = no), cough count on tracheal stimulation 1 (1 = severe, 0 = else), cough count on tracheal stimulation 2 (1 = moderate, 0 = else).

Factors influencing the severity of cough before treatment are summarized in Table 4. The severity of the cough was assessed according to the criteria listed in Table 1, and the baseline pretreatment cough scores are shown in Table 5. The analysis revealed that the location and severity of collapse on fluoroscopy, comorbidities (MMVD and PH), right and left CLLH, BC, cough count on tracheal stimulation, presence of tracheal kinking, and number of collapsed regions on fluoroscopy were not significantly associated with the pretreatment cough scores. Although the *p*-value for left CLLH was <0.05, the regression model was not statistically significant at the 5% significance level (*F* = 1.125, *p* = 0.350). Thus, the right and left CLLH levels were not related to the pretreatment cough score.

**Table 4:** Factors influencing cough severity before treatment.

	Precough score		
	*B*	*β*	*p*
(Constant)	3.995		<0.001^**^
Cervical	−0.061	−0.072	0.667
Thoracic inlet	0.157	0.214	0.093
Intra thoracic	0.063	0.089	0.587
Carina	−0.078	−0.091	0.506
Comorbidity (MMVD)^a^	0.053	0.032	0.789
Comorbidity (PH)^a^	−0.041	−0.022	0.855
Rt CLLH	−0.391	−0.344	0.057
Lt CLLH	0.473	0.452	0.013^*^
Bronchial collapse^a^	−0.001	0.000	0.997
Cough count on tracheal stimulation 1^a^	0.171	0.084	0.460
Cough count on tracheal stimulation 2^a^	0.040	0.023	0.841
Tracheal kinking	0.087	0.048	0.689
Number of collapse regions	0.052	0.041	0.806
F (*p*)	1.125 (0.350)		
Adjusted *R*^2^	0.016		

CLLH, cervical lung lobe herniation; Lt, left; MMVD, myxomatous mitral valve disease; PH, pulmonary hypertension; Rt, right. ^*^p < 0.05 and ^**^p < 0.01.

a Dummy variable: comorbidity (MMVD) (1 = yes, 0 = no), comorbidity (PH) (1 = yes, 0 = no), bronchial collapse (1 = yes, 0 = no), cough count on tracheal stimulation 1 (1 = severe, 0 = else), cough count on tracheal stimulation 2 (1 = moderate, 0 = else).

**Table 5:** Changes in cough before and after treatment.

	Baseline (*n* = 90)	After treatment (*n* = 90)	*p*
Presence of coughing	90 (100.0%)	81 (90.0%)	
*Time of day*			
Dog coughs during the night/early morning	46 (56.8%)	73 (90.1%)	<0.001^**^
Dog coughs continuously during the day	35 (43.2%)	8 (9.9%)	
Subtotal	81 (100.0%)	81 (100.0%)	
*Situation*			
Dog coughs only during excitement	56 (69.1%)	76 (93.8%)	<0.001^**^
Dog coughs at rest during the daytime	25 (30.9%)	5 (6.2%)	
Subtotal	81 (100.0%)	81 (100.0%)	
*Change in cough after treatment*			
Cough disappeared	—	9 (10.0%)	
Cough decreased but remained	—	69 (76.7%)	
Unchanged or increased	—	12 (13.3%)	

^*^p < 0.05 and ^**^p < 0.01.

The type and number of medications prescribed were determined based on the patient’s clinical symptoms on the day of TC diagnosis. Prescription histories of the dogs are summarized in Table 6.

**Table 6:** Prescription history and medication dosage on the day diagnosed with tracheal collapse.

Number of medications used	Treatment	*n* (%)
None (*n* = 26)	Weight loss, humidification, elimination of respiratory irritant	26 (24.0%)
1 medication (*n* = 46)	Bronchodilator: theophylline (10–15 mg/kg PO q12h)	37 (34.2%)
	Anti-inflammatory drug: PDS (0.2–0.25 mg/kg PO q12h)	4 (3.7%)
	Anti-inflammatory drug: fluticasone MDI	1 (0.9%)
	Antitussive drug: codeine (1 mg/kg q12h–2 mg/kg PO q8h)	4 (3.7%)
2 medications (*n* = 27)	Bronchodilator + anti-inflammatory drug: theophylline (10–15 mg/kg q12h) + PDS (0.2–0.25 mg/kg PO q12h)	4 (3.7%)
	Bronchodilator + antitussive drug: theophylline (10–15 mg/kg q12h) + codeine (1 mg/kg q12h–2 mg/kg PO q8h)	19 (17.5%)
	Anti-inflammatory drug + antitussive drug: codeine (2 mg/kg q12h–2 mg/kg PO q8h) + PDS (0.2–0.5 mg/kg PO q12h) or fluticasone MDI	4 (3.7%)
3 medications (*n* = 9)	Bronchodilator + antitussive drug + anti-inflammatory drug: theophylline (7.5–20 mg/kg q12h) + codeine (1 mg/kg q12h–3 mg/kg PO q8h) + PDS (0.25 mg//kg PO q12h–0.35 mg/kg PO q12h)	9 (8.3%)

PDS, prednisolone.

Prescriptions were administered to 108 dogs exhibiting cough symptoms, categorized into four groups based on the number of prescribed medications. The types of drugs prescribed included bronchodilators, anti-inflammatory drugs, and antitussive drugs with detailed maximum and minimum dosages. Twenty-six (24%) patients received no drug prescriptions but were guided on weight management, temperature and humidity control, and the removal of respiratory irritants, such as tobacco smoke. Forty-six dogs (42.5%) were prescribed one type of medication, 27 (25%) received two types, and nine (8.3%) were prescribed three types of medications.

Changes in the timing and circumstances of cough occurrence, as well as the extent of improvement between pretreatment and 2–4 weeks posttreatment, are summarized in Table 5. Despite treatment, 81 dogs (90.0%) continued to cough. The timing and circumstances of cough onset and changes in cough were recorded for these 81 dogs. There was a statistically significant increase in the proportion of coughs occurring at night or early morning from 56.8 to 90.1% (*p* < 0.001). There was also a statistically significant increase from 69.1 to 93.8% (*p* < 0.001) in dogs coughing only when excited. Changes in coughing after treatment were as follows: cough disappearance in 10.0%, reduction but persistence in 76.7%, and similarity or increase in 13.3%.

Changes in the degree of cough improvement according to the number of medications prescribed are summarized in Table 7. At the 5% significance level, there was no statistically significant difference in the changes in cough improvement based on the number of medications prescribed.

**Table 7:** Changes in cough improvement according to the number of prescribed medications.

		Cough disappeared	Cough decreased but remained	Unchanged or increased	Total	*p*
Number of used medications	0	1 (6.3%)	11 (68.8%)	4 (25.0%)	16 (100.0%)	0.224
	1	5 (11.6%)	36 (83.7%)	2 (4.7%)	43 (100.0%)	
	2	3 (12.5%)	17 (70.8%)	4 (16.7%)	24 (100.0%)	
	3	0 (0.0%)	5 (71.4%)	2 (28.6%)	7 (100.0%)	
	Total	9 (10.0%)	69 (76.7%)	12 (13.3%)	90 (100.0%)	

^*^p < 0.05 and ^**^p < 0.01.

## 4 Discussion

This retrospective study provided new insights into the clinical and fluoroscopic characteristics of TC in small-breed dogs. If a middle aged or older small-breed dog exhibits a chronic dry cough, TC should be considered as a differential diagnosis. Radiography and fluoroscopy are commonly used for diagnosing TC, with grading based on the reduction ratio of the tracheal luminal diameter. This study revealed that, in addition to obesity, low BW (i.e., small body size) is also a risk factor for collapse severity. Additionally, the location or grade of collapse observed on fluoroscopy was not statistically related to cough severity. Therefore, a higher grade of collapse should not be assumed to correlate with more severe clinical symptoms. Furthermore, most patients showed a good prognosis and improvement in symptoms after environmental changes and oral medication treatment.

TC is a common cause of chronic coughing in dogs. Previous studies have identified TC in various breeds, including Yorkshire Terriers, Poodles, and Pugs (7, 20, 21, 23, 24). Consistently, the most common breeds in this study were Maltese, Pomeranian, Poodles, and Chihuahua. Although more females than males were affected, there was no significant sex predilection. The age range of dogs diagnosed with TC was 2–19 years, with 101 dogs (91.8%) aged >8 years old. This aligns with findings from other studies that also reported a higher prevalence in older dogs (1, 5, 20, 23). Histological analysis of tracheal cartilage rings from dogs with TC revealed a less homogeneous cartilage matrix with reduced levels of chondroitin sulfate and calcium and fewer chondrocytes compared with those from toy breed dogs with normal tracheas (25, 26). This suggests that TC may be a degenerative condition occurring primarily in middle-aged and older dogs. Concurrent obesity, a factor supported by this study, has also been reported in dogs with TC (7, 20, 27), with most dogs (97.2%) demonstrating a BCS greater than the ideal value of ≥4.

Among the patients, chronic cough was confirmed in all except for two. Additionally, 5.5–10% of dogs with TC had symptoms such as cyanosis, dyspnea, exercise intolerance, and syncope. More than half of the patients had MMVD. Research has shown that small dogs may experience coughing when the left atrium enlarges due to their relatively larger heart and shorter distance from the heart to the thoracic vertebrae, causing compression of the trachea (28). However, a previous study found no association between left atrial enlargement and airway collapse in dogs with MMVD, suggesting that other factors, such as airway inflammation, might be attributable to the cough (4). Obstructive upper respiratory diseases like TC often lead to the secondary development of PH (19, 29). Unlike a previous study where 40% of dogs with bronchomalacia had confirmed PH (29), only 26.4% of dogs with TC in this study had PH; this was potentially underestimated as not all patients underwent echocardiography. Comorbidities did not correlate with the severity of the cough or the grade of collapse. However, given the frequent diagnosis of MMVD and PH in dogs with TC, we recommend that all dogs with a high clinical suspicion of TC undergo echocardiography to screen for MMVD and PH.

The TC grades by region on fluoroscopy were compared among Maltese, Pomeranians, Poodles, Chihuahuas, and other breeds. The collapse was least severe in the cervical region, and most severe in the carinal region across all breeds. Pomeranians exhibited a similar grade of collapse at all three sites, excluding the cervical region, demonstrating a higher severity of collapse at the thoracic inlet and intrathoracic region compared with other breeds. The overall trend showed a natural tapering from the cervical (largest diameter) to the carina (smallest diameter) region (23, 30). Anatomically, the thinning of the cartilage at the ventral midpoint and tracheal muscle from the cranial cervical region to the intrathoracic trachea may be a contributing factor (31). In healthy dogs, the tracheal diameter can be reduced by up to 20% in the intra-thoracic region and 18.6% in the thoracic region on expiratory computed tomography (32). In this study, grade 1 TC was most frequently identified in the cervical region, suggesting that a 25% collapse threshold may result in false positive diagnoses of TC (32, 33). Thus, additional research is needed to compare fluoroscopic features across breeds with adequate sample sizes. Furthermore, consistent with studies indicating that static radiography tends to underdiagnose TC and underestimate its grade compared with fluoroscopy (5, 20), only 64.1% of dogs had TC confirmed by radiography in this study. Thus, the presence of a predisposition to TC on radiography was not significantly related to the TC grade on fluoroscopy.

CLLH is defined as a protrusion of the lung parenchyma beyond the boundaries of the thoracic cavity (34). In a preceding study involving 51 dogs with TC, 70.6% were reported to have CLLH, which was not related to the severity of coughing (23). Similarly, in this study, right and left CLLH occurred in 76.3 and 80% of dogs with TC, respectively, and there was no relationship with the severity of coughing. Although previous studies have reported no association between dog breed or chest shape and CLLH (34), our results showed that Pomeranians had the highest incidence of CLLH (84%) among dogs with TC. Notably, in all breeds, the left CLLH grade was more severe than the right. We speculate that this might be due to the anatomical position of the left lung lobe, which is more anterior than the right lung lobe (21, 35).

Dog positioning might also play a major role in the interpretation of results from fluoroscopy. In this study, all dogs were imaged in the right lateral recumbent position, which may have resulted in an underestimation of the right CLLH compared with the left side (5). Thus, additional studies should include fluoroscopic imaging in left lateral recumbency for comparison.

Mainstem BC is defined as the static or dynamic collapse of the main bronchus on the left or right side (26, 36). In this study, BC was identified in 75 dogs (68.1%) diagnosed with TC via fluoroscopic imaging. Maltese had the highest incidence of BC, whereas Chihuahuas had the lowest. The etiology of BC is unknown; however, it has been posited that the cartilaginous defect in dogs with TC may extend distally to affect the bronchi in some cases (1, 37). In three previous studies where dogs underwent fluoroscopy, tracheal kinking was observed in 27–29.3% of cases (21, 23, 34). In the present study, tracheal kinking was confirmed in 29% (32 dogs) of the dogs with TC and was not associated with the severity of coughing. Although weakening of the cartilage due to TC and increased airway resistance are considered causes of tracheal kinking (23), further research is needed to understand why BC and tracheal kinking frequently occur in patients with TC.

The factors influencing the degree of TC observed on fluoroscopy were examined. Obesity is a well-known risk factor for TC development. Research has shown that by limiting thoracic movement and chest wall compliance, the accumulation of intrathoracic adipose tissue can reduce respiratory function (8). Furthermore, neutering was statistically associated only with the collapse severity in the carina region. Neutered animals exhibited a higher likelihood of obesity than intact animals (38). We hypothesize that the increased BCS due to neutering contributes to the statistical association of the carina demonstrating the highest collapse severity. Consistent with previous studies, left CLLH was exclusively associated with collapse severity in the intrathoracic region. The increased airflow resistance resulting from airway collapse, which hinders pressure relief through coughing and elevates intrathoracic pressure, is believed to be the cause of CLLH (34).

Previous research utilizing bronchoscopy to diagnose TC has also indicated that low BW is associated with more severe airway collapse (1), corroborating our findings. However, when dogs are stratified by BW, it remains unclear why TC predominantly affects small-breed dogs compared with large-breed dogs. The tracheal cross-sectional diameter diminishes as BW decreases (32). According to Poiseuille’s law, resistance is inversely proportional to the fourth power of the airway radius (24). Increased resistance can be mitigated by either enhanced respiratory effort or prolonged inspiratory time, leading to decreased airflow (39). In prior research employing the tidal breathing flow-volume loop, dogs with TC exhibited longer inspiratory times than healthy dogs (24). Consequently, smaller dogs may be more susceptible to TC due to greater resistance stemming from smaller tracheal diameters.

Moreover, in humans, the higher incidence of TMB in children is partly due to the membranous trachea constituting a larger proportion of the trachea than the cartilaginous trachea (40). Small breeds are known to possess a greater proportion of dorsal tracheal membrane than large breeds (41). Additionally, the transverse/longitudinal diameter ratio at the T4 vertebral level is lower in small dogs (28). These anatomical characteristics of the trachea in small dogs are thought to heighten the risk of TC; however, further studies are required to validate these hypotheses.

Although multiple locations were assessed by fluoroscopy, neither the anatomical location nor degree of collapse observed correlated with cough severity. This finding contradicts the hypothesis that tracheal diameter reduction due to TC would increase airway resistance, thereby exacerbating cough. This suggests that tracheal anatomical alterations are not strongly associated with cough severity. The etiology of coughing is complex and multifactorial, encompassing mucosal irritation, inflammation, and turbulent airflow resulting from tracheal narrowing. Thus, further studies are warranted to elucidate the factors precipitating cough in patients with TC.

In this study, BC, CLLH, tracheal kinking, and the number of coughs elicited by tracheal stimulation did not correlate with cough severity. The craniocaudal extent of TC, expressed as the number of affected regions, also showed no association with cough severity. Tracheal manipulation often induces coughing in dogs with airway diseases. Since tracheal palpation is performed on the cervical region, which exhibits the least severe collapse, it may not accurately reflect the overall cough severity for the entire trachea. Given that tracheal palpation has limited diagnostic value and may induce paroxysmal coughing in some patients, we considered that it may not be essential for patients with TC.

In most cases (86.6%), chronic cough improved with weight reductions, environmental changes, and pharmacotherapy. Studies have shown that 71–93% of dogs respond favorably to medical management over a 12-month period, with 50% able to taper off medication (8, 27, 42). Posttreatment, both the frequency and timing of coughing episodes showed statistically significant improvement. However, the number of prescribed medications did not correlate with the degree of improvement. In dogs with TC, coughing occurs primarily during situations of excitement as well as during eating and drinking. This is because the act of swallowing food can stimulate the airway and induce coughing. Therefore, in this study, coughing while eating and coughing, regardless of the situation, were given different scores when assessing cough severity. Additionally, it is important to observe the dog’s condition during meals to evaluate the treatment response.

Among the 26 dogs not prescribed medication for mild cough symptoms, only two dogs lost >2% of their BW 4–6 weeks postdiagnosis; both dogs exhibited improved clinical signs. Despite weight reduction being a crucial treatment for TC (6), most owners find it challenging to achieve weight reduction in their pets. Thus, further research is necessary to investigate the relationship between long-term weight reduction and the amelioration of cough symptoms.

Of the 90 dogs studied, 12 exhibited similar or increased coughing during the initial evaluation of treatment response postdiagnosis. Two patients managed by weight reduction and environmental modification (rather than medication) continued to exhibit mild cough symptoms, and no additional medications were prescribed. One patient showed symptomatic improvement following home nebulization, whereas three patients improved after an increased dosage of oral medication. Six dogs did not return for follow-up, precluding monitoring of their treatment responses.

This study has some limitations that should be acknowledged. One notable limitation is the relatively small and diverse sample of dog breeds evaluated. Additionally, due to the retrospective nature of the research, clinical symptoms were documented in electronic medical records by various veterinarians, and the cough score was subsequently reassessed by two internists. Prospective studies using clinical symptom surveys, like those used in human medicine, could enhance accuracy. Because of the risks associated with anesthesia, tracheal wash or cytobrush cytology was not performed. However, none of the 110 patients showed signs of tracheitis on thoracic radiographs. Furthermore, bronchoscopy has long been regarded as the gold standard for documenting airway collapse in both humans and dogs (15–17). However, in this study, all dogs were diagnosed with TC using fluoroscopy, with neither bronchoscopy nor bronchoalveolar lavage performed. Although bronchoscopy has not been utilized to directly assess the degree of collapse, the high correlation between fluoroscopic imaging during coughing and bronchoscopy findings in previous studies suggests that fluoroscopic imaging is a non-invasive and useful alternative for assessing TC (4, 5).

In conclusion, TC should be considered in the differential diagnosis for chronic cough in small-breed dogs. Smaller, older, and obese dogs are at an increased risk for TC. Contrary to our expectation, fluoroscopic grading during coughing exhibited a poor correlation with cough severity. Consequently, further studies are needed to identify the factors contributing to TC in dogs with chronic cough.

## Data availability statement

The original contributions presented in the study are included in the article/Supplementary material, further inquiries can be directed to the corresponding author.

## Ethics statement

Ethical approval was not required for the studies involving animals in accordance with the local legislation and institutional requirements because I declare that ethical approval is not required as this is a retrospective study using only medical records. Written informed consent was obtained from the owners for the participation of their animals in this study.

## Author contributions

M-RK: Conceptualization, Data curation, Formal analysis, Investigation, Methodology, Software, Visualization, Writing – original draft, Writing – review & editing. S-HK: Conceptualization, Methodology, Supervision, Writing – review & editing. M-OR: Methodology, Supervision, Writing – review & editing. H-YY: Supervision, Writing – review & editing. J-HC: Investigation, Methodology, Writing – review & editing. K-WS: Conceptualization, Methodology, Project administration, Supervision, Writing – review & editing.

## References

[1] Johnson LR Pollard RE. Tracheal collapse and Bronchomalacia in dogs: 58 cases (7/2001–1/2008). J Vet Intern Med. (2010) 24:298–305. doi: 10.1111/j.1939-1676.2009.0451.x, PMID: 20051001

[2] Tangner CH Hobson HP. A retrospective study of 20 surgically managed cases of collapsed trachea. Vet Surg. (1982) 11:146–9. doi: 10.1111/j.1532-950X.1982.tb00691.x

[3] Adamama-Moraitou KK Pardali D Day MJ Prassinos NN Kritsepi-Konstantinou M Patsikas MN. Canine bronchomalacia: a clinicopathological study of 18 cases diagnosed by endoscopy. Vet J. (2012) 191:261–6. doi: 10.1016/j.tvjl.2010.11.021, PMID: 21177126

[4] Singh MK Johnson LR Kittleson MD Pollard RE. Bronchomalacia in dogs with myxomatous mitral valve degeneration. J Vet Intern Med. (2012) 26:312–9. doi: 10.1111/j.1939-1676.2012.00887.x, PMID: 22332787

[5] Johnson LR Singh MK Pollard RE. Agreement among radiographs, fluoroscopy and bronchoscopy in documentation of airway collapse in dogs. J Vet Intern Med. (2015) 29:1619–26. doi: 10.1111/jvim.13612, PMID: 26365563 PMC4895679

[6] Ettinger SJ Kantrowitz B Brayley K. Diseases of the trachea and small airways In: Ettinger SJ Feldman EC, editors. Textbook of veterinary internal medicine. 8th ed. Philadelphia, PA: WB Saunders (2010). 2697–702.

[7] Buback JL Boothe HW Hobson HP. Surgical treatment of tracheal collapse in dogs: 90 cases (1983–1993). J Am Vet Med Assoc. (1996) 208:380–4. doi: 10.2460/javma.1996.208.03.380, PMID: 8575969

[8] Tappin SW. Canine tracheal collapse. J Small Anim Pract. (2016) 57:9–17. doi: 10.1111/jsap.12436 26780854

[9] Spodnick GJ Nwadike BS. Surgical management of extrathoracic tracheal collapse in two large-breed dogs. J Am Vet Med Assoc. (1997) 211:1545–8. doi: 10.2460/javma.1997.211.12.1545, PMID: 9412681

[10] Mims HL Hancock RB Leib MS Waldron DR. Primary tracheal collapse in a cat. J Am Anim Hosp Assoc. (2008) 44:149–53. doi: 10.5326/0440149, PMID: 18451074

[11] Maggiore AD. An update on tracheal and airway collapse in dogs. Vet Clin North Am Small Anim Pract. (2020) 50:419–30. doi: 10.1016/j.cvsm.2019.11.003, PMID: 31864678

[12] Done SH Clayton-Jones DG Price EK. Tracheal collapse in the dog: a review of the literature and report of two new cases. J Small Anim Pract. (1970) 11:743–50. doi: 10.1111/j.1748-5827.1970.tb05579.x, PMID: 5531154

[13] Kamata S Usui N Sawai T Nose K Kitayama Y Okuyama H. Pexis of the great vessels for patients with tracheobronchomalacia in infancy. J Pediatr Surg. (2000) 35:454–7. doi: 10.1016/S0022-3468(00)90213-6, PMID: 10726688

[14] O’Brien JA Buchanan JW Kelly DF. Tracheal collapse in the dog. Vet Radiol. (1966) 7:12–20. doi: 10.1111/j.1740-8261.1966.tb01058.x

[15] Heyer CM Nuesslein TG Jung D Peters SA Lemburg SP Rieger CHL. Tracheobronchial anomalies and stenoses: detection with low-dose multidetector CT with virtual tracheobronchoscopy—comparison with flexible tracheobronchoscopy. Radiology. (2007) 242:542–9. doi: 10.1148/radiol.2422060153, PMID: 17255423

[16] Rudman DT Elmaraghy CA Shiels WE Wiet GJ. The role of airway fluoroscopy in the evaluation of stridor in children. Arch Otolaryngol Head Neck Surg. (2003) 129:305–9. doi: 10.1001/archotol.129.3.305, PMID: 12622539

[17] Doshi J Krawiec ME. Clinical manifestations of airway malacia in young children. J Allergy Clin Immunol. (2007) 120:1276–8. doi: 10.1016/j.jaci.2007.09.048, PMID: 18073123

[18] Keene BW Atkins CE Bonagura JD Fox PR Häggström J Fuentes VL. ACVIM consensus guidelines for the diagnosis and treatment of myxomatous mitral valve disease in dogs. J Vet Intern Med. (2019) 33:1127–40. doi: 10.1111/jvim.15488, PMID: 30974015 PMC6524084

[19] Reinero C Visser LC Kellihan HB Masseau I Rozanski E Clercx C. ACVIM consensus statement guidelines for the diagnosis, classification, treatment, and monitoring of pulmonary hypertension in dogs. J Vet Intern Med. (2020) 34:549–73. doi: 10.1111/jvim.15725, PMID: 32065428 PMC7097566

[20] Macready DM Johnson LR Pollard RE. Fluoroscopic and radiographic evaluation of tracheal collapse in dogs: 62 cases (2001–2006). J Am Vet Med Assoc. (2007) 230:1870–6. doi: 10.2460/javma.230.12.1870, PMID: 17571993

[21] Lee J Yun S Lee I Choi M Yoon J. Fluoroscopic characteristics of tracheal collapse and cervical lung herniation in dogs: 222 cases (2012–2015). J Vet Sci. (2017) 18:499–505. doi: 10.4142/jvs.2017.18.4.499, PMID: 28057909 PMC5746443

[22] Hori Y Nakamura K Kanno N Hitomi M Yamashita Y Hosaka S eds. Effects of the angiotensin-converting enzyme inhibitor alacepril in dogs with mitral valve disease. J Vet Med Sci. (2018) 80:1212–8. doi: 10.1292/jvms.17-0557, PMID: 29937457 PMC6115264

[23] Jung D-Y Park S-M Lim G-H Seo K-W Oh Y-I Youn H-Y. Assessment of MMP-9 and clinical characteristics in dogs with tracheal collapse based on cough severity and fluoroscopic findings: a cross-sectional study. BMC Vet Res. (2024) 20:52. doi: 10.1186/s12917-023-03872-1, PMID: 38341543 PMC10858467

[24] Pardali D Adamama-Moraitou KK Rallis TS Raptopoulos D Gioulekas D. Tidal breathing flow-volume loop analysis for the diagnosis and staging of tracheal collapse in dogs. J Vet Intern Med. (2010) 24:832–42. doi: 10.1111/j.1939-1676.2010.0513.x, PMID: 20412439

[25] Dallman MJ McClure RC Brown EM. Histochemical study of normal and collapsed tracheas in dogs. Am J Vet Res. (1988) 49:2117–25. PMID: 2467593

[26] Reinero CR Masseau I. Lower airway collapse: revisiting the definition and clinicopathologic features of canine bronchomalacia. Vet J. (2021) 273:105682. doi: 10.1016/j.tvjl.2021.105682, PMID: 34148610

[27] White RAS Williams JM. Tracheal collapse in the dog - is there really a role for surgery? A survey of 100 cases. J Small Anim Pract. (1994) 35:191–6. doi: 10.1111/j.1748-5827.1994.tb01685.x

[28] Uehara T Orito K Fujii Y. CT-based anatomical features of large airway and heart volume in dogs of different body size. Vet J. (2019) 246:21–6. doi: 10.1016/j.tvjl.2019.01.014, PMID: 30902185

[29] Gamracy J Wiggen K Vientós-Plotts A Reinero C. Clinicopathologic features, comorbid diseases, and prevalence of pulmonary hypertension in dogs with bronchomalacia. J Vet Intern Med. (2022) 36:417–28. doi: 10.1111/jvim.16381, PMID: 35129853 PMC8965257

[30] Violette NP Weisse C Berent AC Lamb KE. Correlations among tracheal dimensions, tracheal stent dimensions, and major complications after endoluminal stenting of tracheal collapse syndrome in dogs. J Vet Intern Med. (2019) 33:2209–16. doi: 10.1111/jvim.15555, PMID: 31290188 PMC6766514

[31] Dabanoğlu I Öcal MK Kara ME. A quantitative study on the trachea of the dog. Anat Histol Embryol. (2001) 30:57–9. doi: 10.1046/j.1439-0264.2001.00301.x, PMID: 11284164

[32] Leonard CD Johnson LR Bonadio CM Pollard RE. Changes in tracheal dimensions during inspiration and expiration in healthy dogs as detected via computed tomography. Am J Vet Res. (2009) 70:986–91. doi: 10.2460/ajvr.70.8.986, PMID: 19645579

[33] Yoon H Yu J An G Bang S Kwon D Kim H. CT and radiographic evaluation of bronchial collapsibility at forced expiration in asymptomatic brachycephalic dogs. Vet Radiol Ultrasound. (2020) 61:167–80. doi: 10.1111/vru.12829, PMID: 31896169

[34] Nafe LA Robertson ID Hawkins EC. Cervical lung lobe herniation in dogs identified by fluoroscopy. Can Vet J. (2013) 54:955–9. PMID: 24155415 PMC3781426

[35] Evans HE de Lahunta A. The respiratory system In: Miller’s anatomy of the dog. 4th ed. Louis, MO: Elsevier Saunders Inc. (2013). 338–60.

[36] Kelly D Juvet F Lamb V Holdsworth A. Bronchial collapse and bronchial stenting in 9 dogs. J Vet Intern Med. (2023) 37:2460–7. doi: 10.1111/jvim.16859, PMID: 37695258 PMC10658526

[37] Johnson L. Tracheal collapse: diagnosis and medical and surgical treatment. Vet Clin North Am Small Anim Pract. (2000) 30:1253–66. doi: 10.1016/S0195-5616(00)06005-8 11221980

[38] Bjørnvad CR Gloor S Johansen SS Sandøe P Lund TB. Neutering increases the risk of obesity in male dogs but not in bitches—a cross-sectional study of dog- and owner-related risk factors for obesity in Danish companion dogs. Prev Vet Med. (2019) 170:104730. doi: 10.1016/j.prevetmed.2019.104730 31421500

[39] Rozanski EA Greenfield CL Alsup JC McKiernan BC Hungerford LL. Measurement of upper airway resistance in awake untrained dolichocephalic and mesaticephalic dogs. Am J Vet Res. (1994) 55:1055–9. doi: 10.2460/ajvr.1994.55.08.1055, PMID: 7978643

[40] Boogaard R Huijsmans SH Pijnenburg MWH Tiddens HAWM de Jongste JC Merkus PJFM. Tracheomalacia and bronchomalacia in children: incidence and patient characteristics. Chest. (2005) 128:3391–7. doi: 10.1378/chest.128.5.3391 16304290

[41] Lindl Bylicki BJ Johnson LR Pollard RE. Comparison of the radiographic and tracheoscopic appearance of the dorsal tracheal membrane in large and small breed dogs. Vet Radiol Ultrasound. (2015) 56:602–8. doi: 10.1111/vru.12276, PMID: 26173473

[42] Ayres SA Holmberg DL. Surgical treatment of tracheal collapse using pliable total ring prostheses: results in one experimental and 4 clinical cases. Can Vet J. (1999) 40:787–91. PMID: 10563237 PMC1540003

