# Supplementary material for: A retrospective study of tracheal collapse in small-breed dogs: 110 cases (2022–2024)
Source: Front Vet Sci. 2024 Aug 14;11:1448249. doi: 10.3389/fvets.2024.1448249 (PMC11349713; doi:10.3389/fvets.2024.1448249)
Supplement: Supplementary file 1 [file Table_1.docx]

Supplementary Material

**Supplementary Table 1.** Correlations among age, BW, BCS, CLLH, and cough count on tracheal stimulation in dogs with TC

|  | Age | Weight | BCS | Rt CLLH | Lt CLLH | Cough count on tracheal stimulation |
| --- | --- | --- | --- | --- | --- | --- |
| Age | 1 |  |  |  |  |  |
| BW | .110 | 1 |  |  |  |  |
| BCS | -.148 | .301* | 1 |  |  |  |
| Rt CLLH | .057 | -.040 | .021 | 1 |  |  |
| Lt CLLH | .087 | .009 | .069 | .797** | 1 |  |
| Cough count on tracheal stimulation | .044 | -.036 | -.037 | .082 | -.035 | 1 |

* *p* < .05, ** *p* < .01

BCS, body condition score; BW, body weight; CLLH, cervical lung lobe herniation; Lt, Left; Rt, Right; TC, tracheal collapse

**Supplementary Table 2.** LA:Ao and VLAS affecting the collapse severity by TC regions

| Variables | Cervical | | | Thoracic inlet | | | Intra thoracic | | | Carina | | |
| --- | --- | --- | --- | --- | --- | --- | --- | --- | --- | --- | --- | --- |
|  | B | β | *p* | B | β | *p* | B | β | *p* | B | β | *p* |
| (Constant) | 1.420 |  | .018* | 3.748 |  | <.001** | 2.371 |  | .004** | 2.993 |  | <.001** |
| LA:Ao | .049 | .024 | .894 | -.058 | -.021 | .905 | .054 | .020 | .912 | .098 | .047 | .795 |
| VLAS | -.320 | -.182 | .314 | -.442 | -.188 | .293 | .059 | .025 | .890 | .018 | .010 | .957 |
| F (*p*) | .834 (.439) | | | 1.275 (.287) | | | .052 (.950) | | | .088 (.916) | | |
| Adjusted R² | -.005 | | | .009 | | | -.032 | | | -.031 | | |
| * *p* < .05, ** *p* < .01 | | | | | | | | | | | | |

Among the 110 TC dogs, LA:Ao and VLAS were recorded for the 62 dogs that underwent echocardiography. Multiple linear regression analysis was used for the statistical evaluation.

LA:Ao, left atrial aortic root ratio; TC, tracheal collapse; VLAS, vertebral left atrial size

🡪 The influence of LA: Ao and VLAS on the severity of tracheal collapse by region is shown in the following Table. At a 5% significance level, the severity of tracheal collapse in all regions was found to be statistically insignificant.

Since the F (p) values were not statistically significant, the model is not considered appropriate. LA:Ao and VLAS are not significant factors in explaining the severity of tracheal collapse by region.

**Supplementary Table 3.** Differences in cough severity before treatment based on the presence of static collapse

| Group | N | Median (IQR) | *p* |
| --- | --- | --- | --- |
| Dogs with static collapse | 61 | 4.00 (1.00) | .404 |
| Dogs without static collapse | 49 | 4.50 (2.00) |  |
| * *p* < .05, ** *p* < .01 | | | |

Based on the fluoroscopic results of 110 TC dogs, the presence of static collapse was evaluated. Since some dogs had both static and dynamic collapse depending on the collapse regions, it was deemed more appropriate to evaluate based on the presence of static collapse rather than classifying patients into static and dynamic collapse categories.

The Mann-Whitney U test was used for statistical analysis. The differences in cough severity before treatment based on the presence of static collapse are shown in the following Table. At a 5% significance level, there were no statistically significant differences in cough severity before treatment according to the presence of static collapse.
